# Supplementary figures and images for: Accuracy of CT perfusion ischemic core volume and location estimation: A comparison between four ischemic core estimation approaches using syngo.via
Source: PLoS One. 2022 Aug 2;17(8):e0272276. doi: 10.1371/journal.pone.0272276 (PMC9345340; doi:10.1371/journal.pone.0272276)

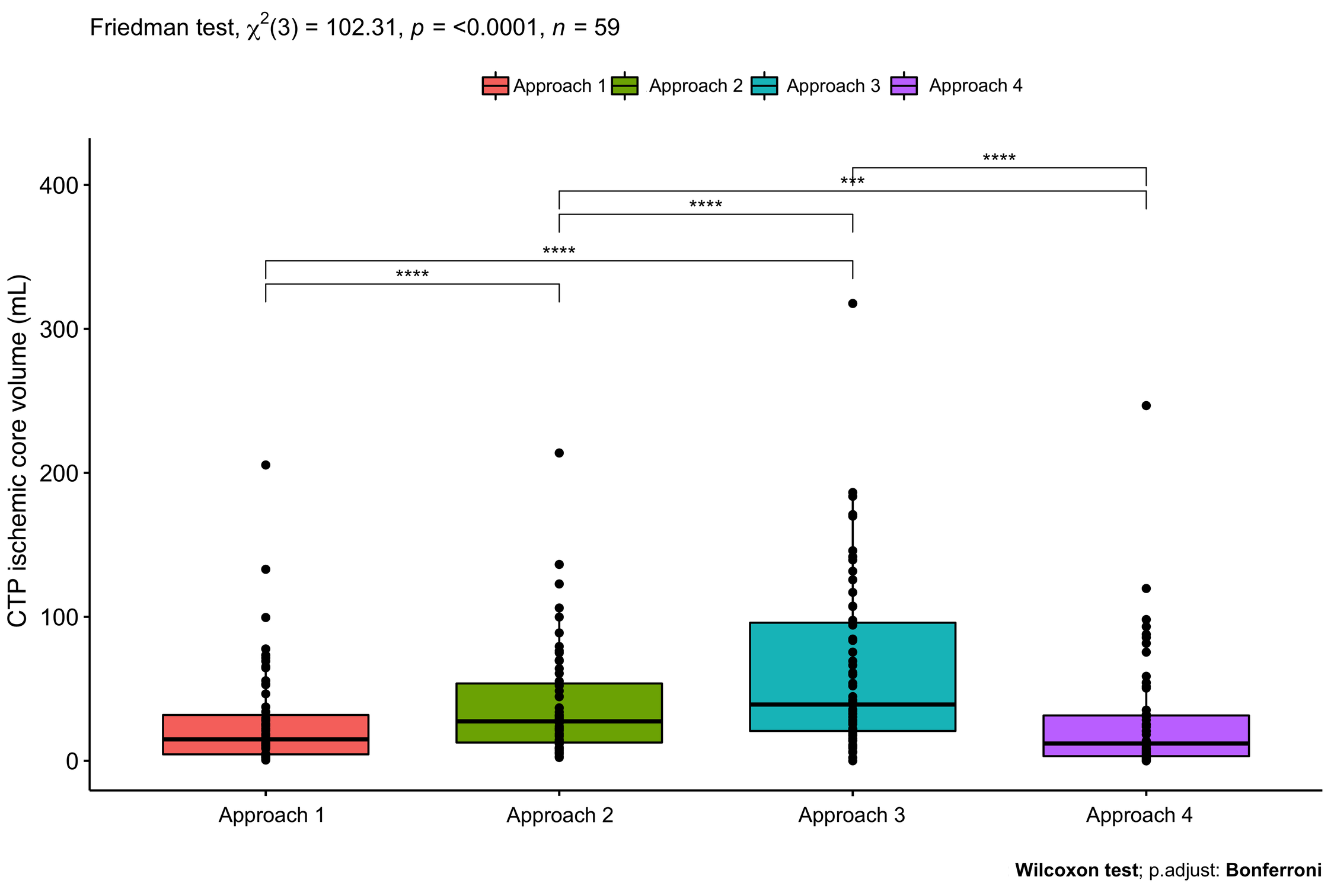

Supplement: S1 Fig — The estimated CTP ischemic core volume was statistically significantly different for the four core estimation approaches using Friedman test, χ2 = 102.31, p<0.001. Pairwise Wilcoxon signed rank test between groups revealed statistically significant differences in CTP ischemic core volume between approach 1-approach 2 (p<0.001), approach 1-approach 3 (p<0.001), approach 2-approach 3 (p<0.001), approach 2-approach 4 (p<0.001), and approach 3-approach 4 (p<0.001). CTP = computed tomography perfusion. (TIF) [file pone.0272276.s001.tif]

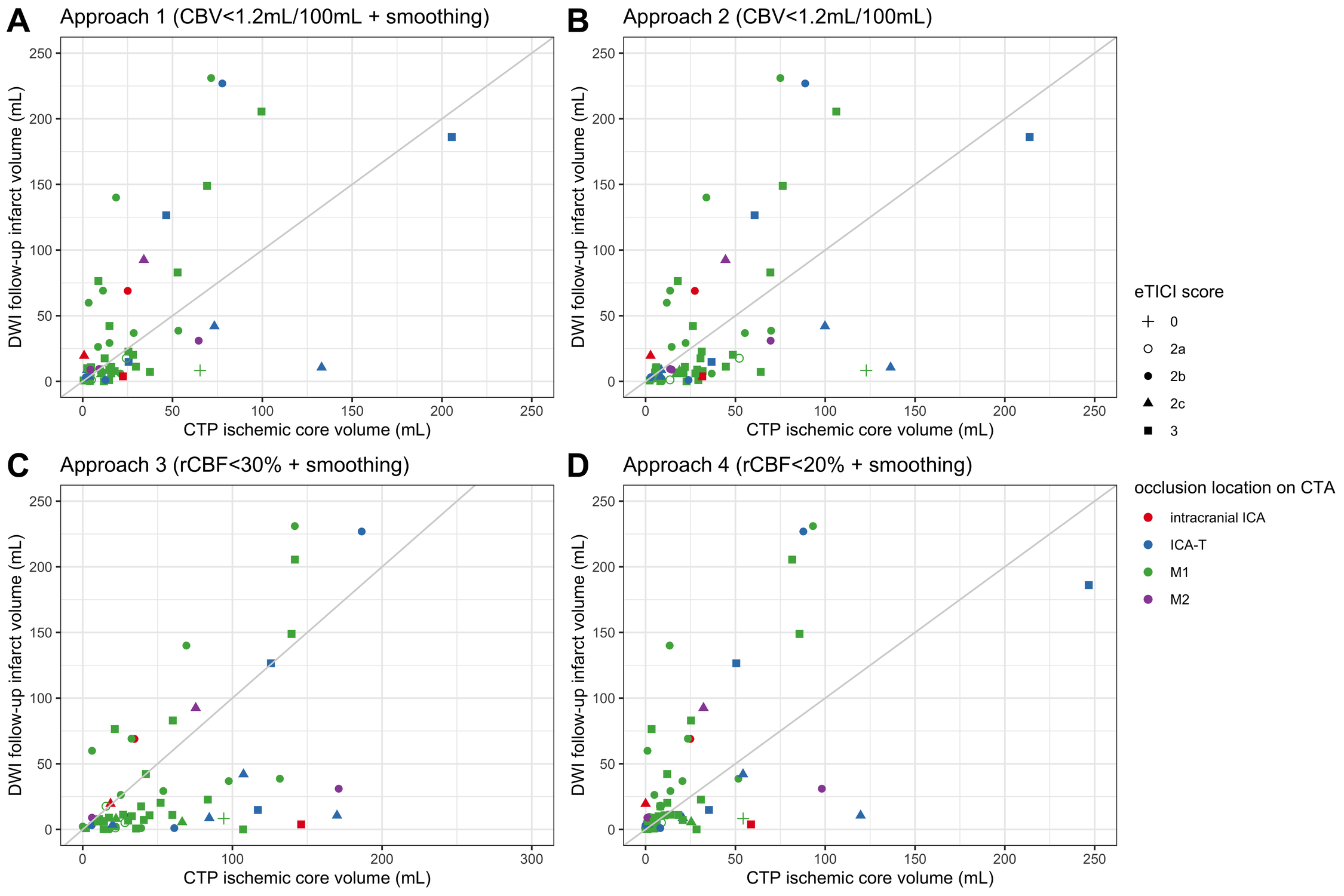

Supplement: S2 Fig — Scatter plots show the agreement between the estimated CTP ischemic core volume and the follow-up DWI infarct lesion for (A) approach 1, (B) approach 2, (C) approach 3, and (D) approach 4. The solid grey line represents the identity line. Points below the identity line (grey) indicate a larger CTP ischemic core volume compared to the follow-up DWI lesion, i.e., overestimation by CTP. Points above the identity indicate underestimation by CTP or infarct growth. CTA = CT angiography; CTP = CT perfusion; DWI = diffusion weighted imaging; ICA = intracranial carotid artery; ICA-T = intracranial carotid artery terminus; M1 = M1 (horizontal) segment of the middle cerebral artery; M2 = M2 (insular) segment of the middle cerebral artery. (TIF) [file pone.0272276.s002.tif]

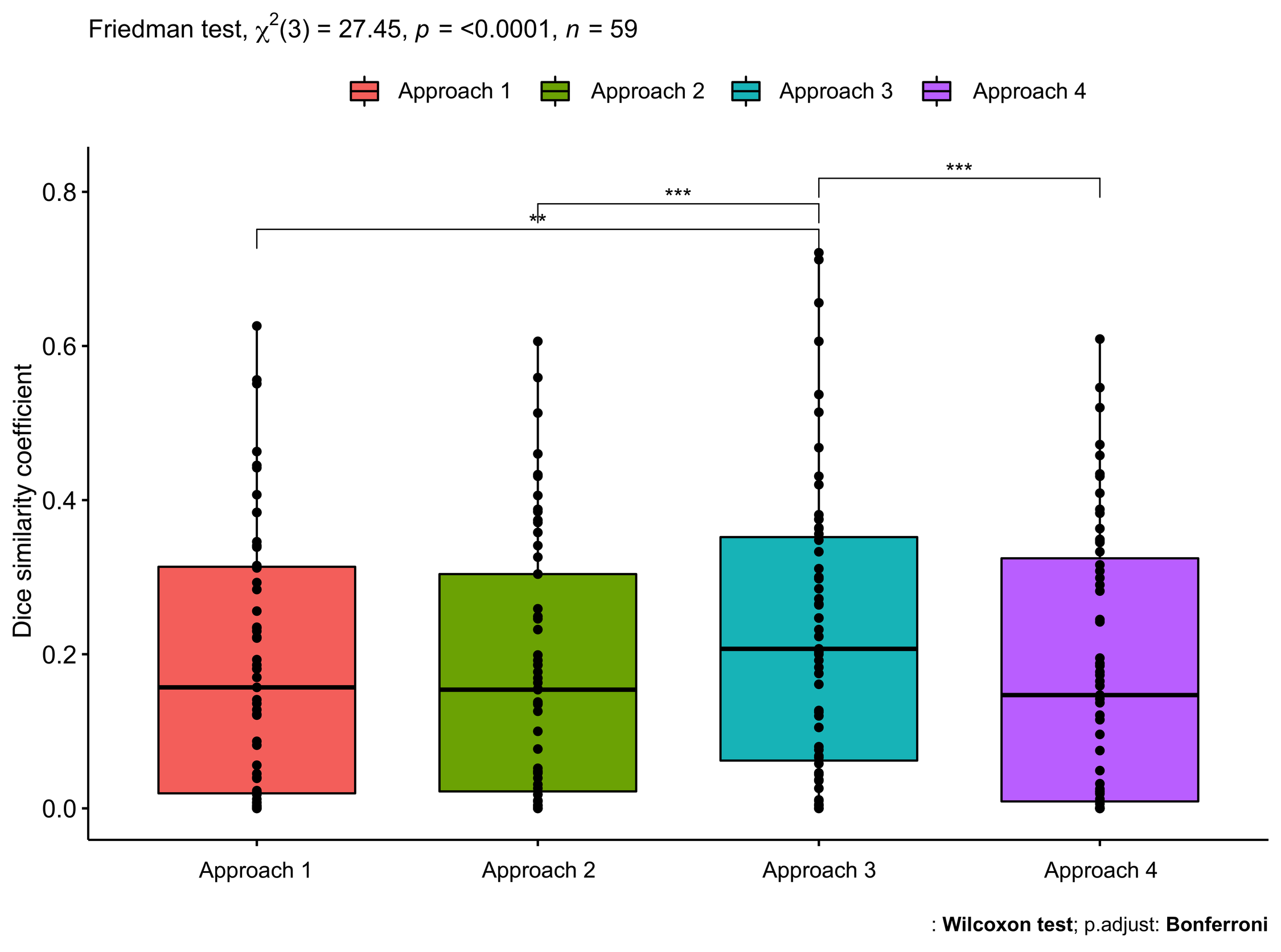

Supplement: S3 Fig — Dice was statistically significantly different for the different approaches using Friedman test, χ2 = 27.45, p<0.001. Pairwise Wilcoxon signed rank test between groups revealed statistically significant differences in Dice between approach 1-approach 3 (p = 0.005), approach 2-approach 3 (p = 0.001), and approach 3-approach 4 (p = 0.001). CTP = computed tomography perfusion. (TIF) [file pone.0272276.s003.tif]

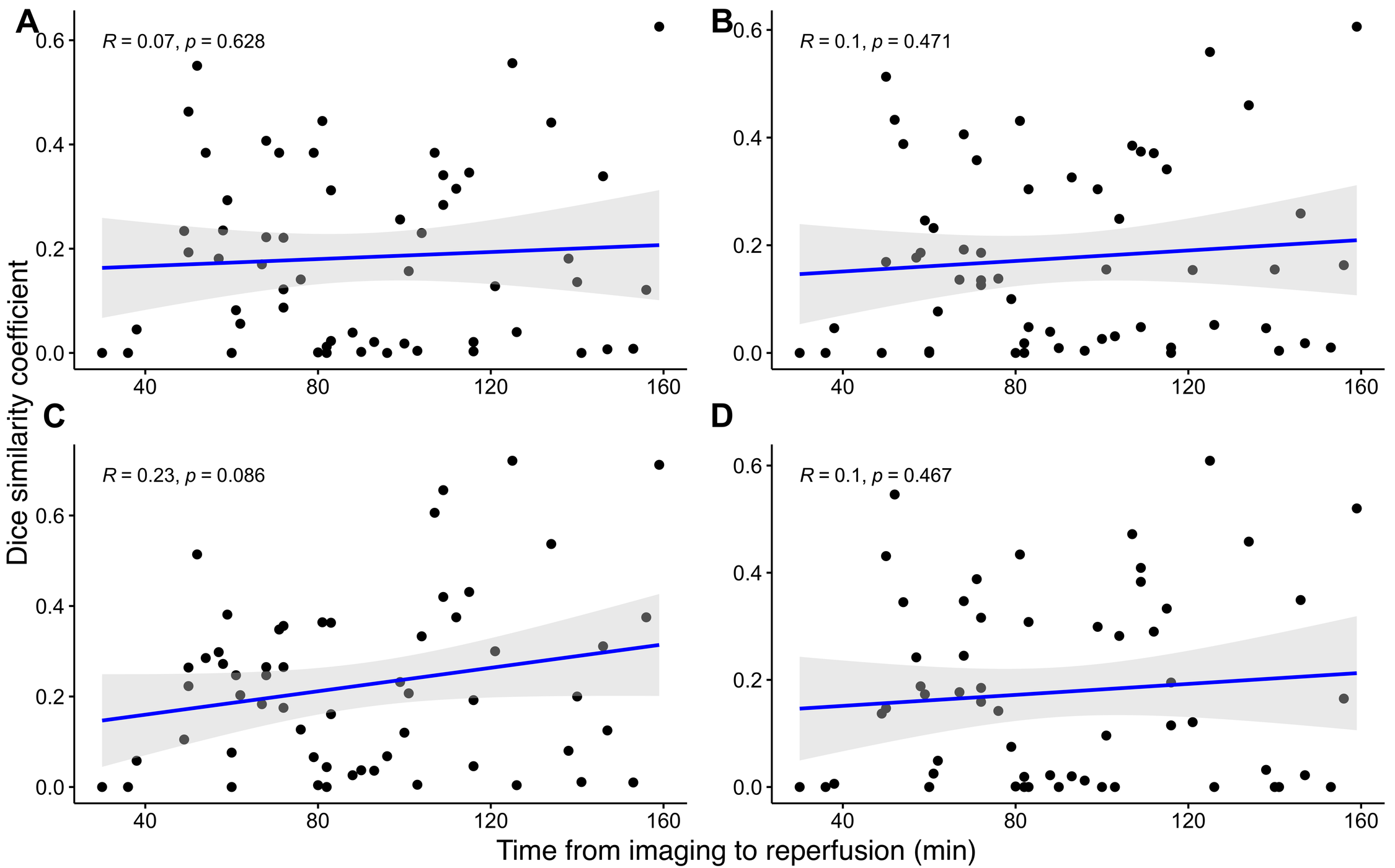

Supplement: S4 Fig — Scatter plots show the association between time from CTP imaging to reperfusion and Dice similarity coefficient for (a) approach 1, (b) approach 2, (c) approach 3, and (d) approach 4. R = Pearson correlation coefficient. (TIF) [file pone.0272276.s004.tif]

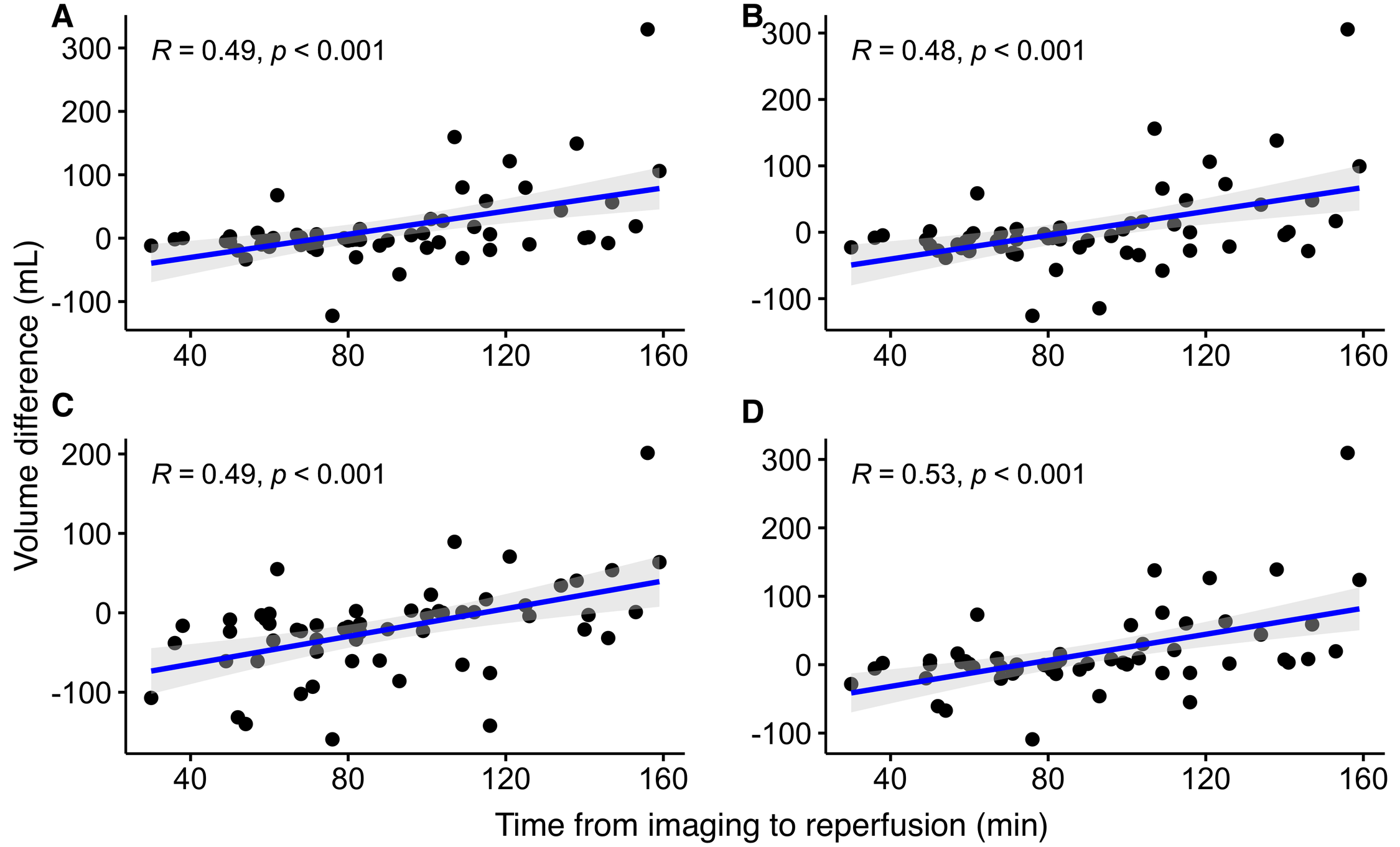

Supplement: S5 Fig — Scatter plots show the association between time from CTP imaging to reperfusion and volume difference for (a) approach 1, (b) approach 2, (c) approach 3, and (d) approach 4. R = Pearson correlation coefficient. (TIF) [file pone.0272276.s005.tif]
