# Supplementary material for: Accuracy of CT perfusion ischemic core volume and location estimation: A comparison between four ischemic core estimation approaches using syngo.via
Source: PLoS One. 2022 Aug 2;17(8):e0272276. doi: 10.1371/journal.pone.0272276 (PMC9345340; doi:10.1371/journal.pone.0272276)
Supplement: Attachment — Submitted filename: Response_letter.docx [file pone.0272276.s006.docx]

Dear Professor Psychogios,

We thank you for the opportunity to improve our manuscript. Revisions are provided below (point-by-point) as well as in a separate file with tracked changes. We are happy to discuss any further changes, if needed.

**Journal Requirements:**

J1. Please ensure that your manuscript meets PLOS ONE's style requirements, including those for file naming. The PLOS ONE style templates can be found at

<https://journals.plos.org/plosone/s/file?id=wjVg/PLOSOne_formatting_sample_main_body.pdf> and <https://journals.plos.org/plosone/s/file?id=ba62/PLOSOne_formatting_sample_title_authors_affiliations.pdf>

Thank you. We have adapted our manuscript, the title page, and file names to meet PLOS ONE’s style requirements.

J2. Thank you for stating the following in the Competing Interests section: 
"HAM is co-founder and shareholder of Nicolab. All other authors report no conflicts of interest. BJE reports grants from Stryker Neurovascular and personal fees from Dekra and from Novartis, outside the submitted work. CBLMM reports grants from TWIN, during the conduct of the study and grants from CVON/Dutch Heart Foundation and from Stryker outside the submitted work (paid to institution) and is shareholder of Nicolab. All other authors did not receive support from any organization for the submitted work, had no financial relationships with any organizations that might have an interest in the submitted work in the previous three years, and had no other relationships or activities that could appear to have influenced the submitted work."

We note that you received funding from a commercial source: Stryker Neurovascular, Dekra, Novartis, TWIN, CVON/Dutch Heart Foundation and Stryker.

J2.1 Please provide an amended Competing Interests Statement that explicitly states this commercial funder, along with any other relevant declarations relating to employment, consultancy, patents, products in development, marketed products, etc.

We have adapted our Competing Interests Statement. (Page 22, line 629-637):

*“We have read the journal’s policy and the authors of this manuscript have the following competing interests: HAM is co-founder and shareholder of Nicolab. BJE reports grants from Stryker Neurovascular and personal fees from Dekra and Novartis outside the submitted work. CBLMM is shareholder of Nicolab and reports grants from TWIN, CVON/Dutch Heart Foundation, and Stryker outside the submitted work. This does not alter our adherence to PLOS ONE policies on sharing data and materials. All other authors declare no support from any organization or financial relationships with any organizations that might have an interest in the submitted work. All authors declare no other relationships or activities that could appear to have influenced the submitted work.”*

J2.2 Within this Competing Interests Statement, please confirm that this does not alter your adherence to all PLOS ONE policies on sharing data and materials by including the following statement: "This does not alter our adherence to PLOS ONE policies on sharing data and materials.” (as detailed online in our guide for authors <http://journals.plos.org/plosone/s/competing-interests>).

We have adapted our Competing Interests Statement accordingly. Please see answer J2.1

J2.3 If there are restrictions on sharing of data and/or materials, please state these. Please note that we cannot proceed with consideration of your article until this information has been declared.

Individual patient data cannot be made available under the Dutch law, as we did not obtain patient approval for sharing individual, coded patient data. Syntax files and output of statistical analyses will be made available upon reasonable request.

We have added minor additions to the data availability statement in our manuscript. (Page 10, line 248-253):

*“Individual patient data cannot be made available under Dutch law as we did not obtain patient approval for sharing individual, coded patient data. In line with privacy regulations, publication of individual patient data as well as syntax files and output of statistical analyses is forbidden by the Data Privacy Officer of the Amsterdam UMC. All syntax files and output of statistical analyses are available on reasonable request. Such requests can be addressed to* [*m.w.a.caan@amsterdamumc.nl*](mailto:m.w.a.caan@amsterdamumc.nl)*.”*

J2.4 Please include your amended Competing Interests Statement within your cover letter. We will change the online submission form on your behalf.

We have included the amended Competing Interests Statement within our cover letter. Thank you for changing the online submission.

J3. We note that you have indicated that data from this study are available upon request. PLOS only allows data to be available upon request if there are legal or ethical restrictions on sharing data publicly. For more information on unacceptable data access restrictions, please see <http://journals.plos.org/plosone/s/data-availability#loc-unacceptable-data-access-restrictions>.

Please see comment J2.3.

In your revised cover letter, please address the following prompts:

J3.1 If there are ethical or legal restrictions on sharing a de-identified data set, please explain them in detail (e.g., data contain potentially sensitive information, data are owned by a third-party organization, etc.) and who has imposed them (e.g., an ethics committee). Please also provide contact information for a data access committee, ethics committee, or other institutional body to which data requests may be sent.

*“Individual patient data cannot be made available under Dutch law as we did not obtain patient approval for sharing individual, coded patient data. In line with privacy regulations, publication of individual patient data as well as syntax files and output of statistical analyses is forbidden by the Data Privacy Officer of the Amsterdam UMC. All syntax files and output of statistical analyses are available on reasonable request. Such requests can be addressed to* [*m.w.a.caan@amsterdamumc.nl*](mailto:m.w.a.caan@amsterdamumc.nl)*.”*

J3.2 If there are no restrictions, please upload the minimal anonymized data set necessary to replicate your study findings as either Supporting Information files or to a stable, public repository and provide us with the relevant URLs, DOIs, or accession numbers. For a list of acceptable repositories, please see <http://journals.plos.org/plosone/s/data-availability#loc-recommended-repositories>.

Please see answer J3.1

J4. Please include your full ethics statement in the ‘Methods’ section of your manuscript file. In your statement, please include the full name of the IRB or ethics committee who approved or waived your study, as well as whether or not you obtained informed written or verbal consent. If consent was waived for your study, please include this information in your statement as well.

Thank you. We have included a full ethics statement to our Methods section. (Page 10, lines 242-245):

*“This study was reviewed by the Medical Ethical Committee Board of the Amsterdam University Medical Centers (location AMC) and informed consent was waived (Reference W19_281#19.334) as retrospective, observational studies do not fall under the scope Medical Research Involving Human Subject Act (WMO).”*

**Reviewer #1:**

This is a well written study, which addresses an important clinical question with merging importance especially, as LVOs are more and more prone to treatment in expanded time windows and different approaches for estimation of penumbra and core assessment are used in the field.

1.1 This already leads to one of my major concerns regarding this work:
- the authors chose very early treatment time points (Mr Clean onset -door: 240min, here 140min; Hermes Onset to random 196min vs 83 onset to image here). Firstly, these are timelines which in my eyes do not apply to most clinical settings, as the o-d times are much longer, especially for shipped patients.

We thank the reviewer for pointing us to this limitation. We agree that the patients in our study cohort received imaging in the hyperacute time window and relatively shortly after symptom onset (median 83 minutes). This could partially be explained by the fact that most patients included in our analysis were directly admitted to the EVT-capable center and did not have to be transferred from a primary stroke center. Another reason could be that most patients who were included in the MR CLEAN-NO IV trial received follow-up DWI imaging and were subsequently included in our analysis. The MR CLEAN-NO IV trial studied the added value of IV alteplase prior to EVT in the 0-4.5h time window.

We have added this as a limitation to our study. (Page 20, line 557-564):

*“Several limitations to our study should be noted. First, all included patients who underwent EVT had relatively small ischemic core volumes (median range 13-40 mL). More specifically, 43/59 (73%) patients received baseline imaging in the hyperacute time window (i.e., within 3 hours after symptom onset) where CTP is not recommended for selection for EVT. The median onset-to-imaging and onset-to-groin times were 83 and 140 minutes, respectively. Therefore, we are not able to draw conclusions about the volumetric or spatial accuracy of syngo.via for large core volumes >70 mL or for patients who presented outside hyperacute time window, for example due to transfer from a mothership hospital.”*

1.2 Secondly, the perfusion approach is not applicable to the timelines studied here, as current guideline recommendation is beyond the 4,5 rep. 6hrs window. Thus it would have made more sense to focus on later time points.
We thank the reviewer for this comment and we acknowledge that CTP is indeed not recommended for selection for IVT or EVT in the 0-4.5, resp. 0-6h time windows. We agree that CTP should currently not be used for patient selection for EVT in the earlier time windows. However, we feel that for diagnostic purposes, it is relevant to quantify the spatial and volumetric accuracy, both for the hyperacute as in the later time windows. We agree that it would have been interesting to include more patients in the later time window. However, we were only able to include patients with follow-up DWI. These follow-up DWI data were mostly acquired in the setting of either the MR CLEAN-NO IV or the MR CLEAN-MED trials which were focusing on patients in the 0-4.5 or 0-6h time window.

We have specified the origin of our study cohort in the Results section and specified that patients received 24h follow-up imaging as part of follow-up imaging from the concerning randomized trial. (Page 11, line 275-279):

*“Most patients (49/59; 83%) in our study cohort were included in one of the randomized controlled trials of the CONTRAST consortium (i.e., MR CLEAN-NO IV, MR CLEAN-MED or MR CLEAN-LATE) [24] and received 24h follow-up DWI as part of the pre-specified follow-up imaging of the concerning trial [25–27].”*

We have addressed the Discussion section (Page 20, line 558-565):

*“More specifically, 43/59 (73%) patients received baseline imaging in the hyperacute time window (i.e., within 3 hours after symptom onset) where CTP is not recommended for selection for EVT. The median onset-to-imaging and onset-to-groin times were 83 and 140 minutes, respectively. Therefore, we are not able to draw conclusions about the volumetric or spatial accuracy of syngo.via for large core volumes >70 mL or for patients who presented outside hyperacute time window, for example due to transfer from a mothership hospital. Of note, for patients who present within this time window, CTP is not recommended for selection for EVT.”*

1.3 Another point is the delay of the MRI, which dependent on TICI success will influence the final DWI lesion size. can the authors comment on that and provide an analysis of 2b and final DWI size compared to 3?
We fully agree with the reviewer and acknowledge that this is a major limitation of our analysis.

We have emphasized this in the limitations section of our Discussion (Page 21, lines 589-593):

*“Fourth, the infarct is likely to expand in the time between CTP acquisition and reperfusion – especially in patients with incomplete micro- or macrovascular reperfusion (i.e., eTICI 2b or 2c) –, which make the degree of reperfusion and timing of the follow-up imaging important factors to consider when performing accuracy assessments [35].”*

Also, we have added an additional sensitivity analysis (“*Sensitivity analysis comparing patients with incomplete vs. complete reperfusion (eTICI 2b vs. eTICI 3*”) between the (newly scored) eTICI 2b vs. eTICI 3 subgroups to the Results section. (Page 16-17, lines 399-481):

We have performed additional analyses on the volumetric (ICC) and spatial (Dice) agreement for these two subgroups and found that for patients with complete reperfusion, all approaches showed moderate-good volumetric agreement and low spatial agreement between the estimated CTP core the 24h follow-up DWI infarct. (Page 16-17, Tables 2-3)

1.4 Another drawback is the use of different MRI field strengths within the cohort (3 vs 1,5T), there is some evidence form stereotactic studies, that there might be great differences between the two field strengths (doi:10.3340/jkns.2014.55.3.136). Can the authors please comment on that?

We thank the reviewer for pointing us to this important issue. We have added this as a limitation to our study in the Discussion section of our manuscript. (Page 21, lines 594-597):

*“Fifth, we included patients with both 1.5 and 3.0 T follow-up DWI scans. As it has been shown that there might be great volumetric and spatial differences between the two field strengths, this could have influenced our results [36].”*

1.5 Another problem could be the non-blinded TICI evaluation, which was done by the interventionalist and might lead to BIAS. It would be worthwhile to crosscheck those by a blinded rater.
We agree that the TICI evaluation by the interventionalist could have introduced bias. Unfortunately, there were no independent core lab observations available at the time of the writing of the manuscript. However, in the meantime, the data of the MR CLEAN-NO IV trials has become available. As most patients included in our analysis were also included in the MR CLEAN-NO IV trial, we could resort to the independent core lab TICI evaluations. These observations were all scored blindly by an independent observer who was part of the Collaborations for New Treatment of Acute Stroke (CONTRAST) consortium core lab. For patients who were not included in the MR CLEAN-NO IV trial or for whom there was no core lab observation available (n=19), the post-EVT DSA images were crosschecked by an independent, blinded rater who is part of the CONTRAST core lab.

We have adapted the specification of the posttreatment imaging assessment this in our manuscript. (Page 7, lines 151-156):

*“Posttreatment recanalization rate was scored as the extended Thrombolysis in Cerebral Infarction (eTICI) score by an independent core lab from the Collaboration for New Treatments of Acute Stroke (CONTRAST) consortium (n=40). For patients not included in one of the CONTRAST trials or for whom there was no core lab observation available (n=19), posttreatment DSAs were evaluated by an independent, blinded observer (>5 years of experience) who is part of the CONTRAST consortium core lab.”*

We have adapted S2 Fig with the updated eTICI scores. (S2 Fig)

1.6 Table 1 shows, that for some metrics not all informations form every patient were available....how did the authors deals with that?

We thank the reviewer for noticing. Indeed, unfortunately, not all clinical baseline variables were available for all patients at the time of writing of the manuscript. However, in the meantime, clinical data from the MR CLEAN-NO IV trial has become available. Since a considerable number of patients included in our analysis were also included in the MR CLEAN-NO IV trial, we were able to add missing clinical baseline variables for patients who were also enrolled in the MR CLEAN-NO IV trial. Although we regret that not all clinical variables are available for all patients included in our analysis, we feel that the data represented in Table 1 still provides an accurate overview of the included population as most patients had baseline data available.

We have updated Table 1 with the updated values. (Table 1)

1.7 Table 1: median follow up DWI time 23min? probably 23hrs...?

Indeed, this should be ‘hours’. We thank the reviewer for noticing and have adapted this accordingly. (Page 11-13, Table 1)

1.8 Line 307 duplication of "from"

Thank you. Corrected.

1.9 Suppl. figure S1 in my eyes does not add visual information, as I can visually barely detect sign. differences between the approaches.

We agree with the reviewer that it is hard to clearly see differences in S1 Fig and now see that this figure has limited added value. Hence, we have decided to remove S1 Fig from the Supporting Information and removed the references to S1 Fig from the manuscript. S2-S6 Figs have been renamed to S1-S5 Figs, accordingly.

**Reviewer #2:**

Overall interesting topic which needs further research as these thresholds are still to be validated and fu infarct volume is a good approximation.

2.1 One reference you might add to the discussion as it looks at a similar topic outlining the discrepancies between different software packages: <https://pubmed.ncbi.nlm.nih.gov/33216157/>

We thank the reviewer for this suggestion and have added the reference to both the Introduction and Discussion section.

(Page 4, line 92-95):

*“The various commercially available CTP post-processing software packages use different approaches based on CBV or relative CBF (rCBF), and Tmax or rCBF parameters thresholds to estimate the respective ischemic core and penumbra, which complicates the generalizability of CTP results [8–11].”*

(Page 19, line 517-519):

*“Another study compared the core estimates from four software packages (i.e., RAPID, VEOcore, syngo.via, and Olea) and found volume differences up to 33 mL between the different software packages [11].”*
